# Design and Implementation of Online Meticulous Painting System Based on Mobile IoT Service

**DOI:** 10.1155/2022/3144255

**Published:** 2022-06-03

**Authors:** Cui Xu

**Affiliations:** Changchun Institute of Science and Technology, Changchun, Jilin 136000, China

## Abstract

Contemporary art is gradually diversifying, and the aesthetics of artists have changed and progressed together with the times. Some brush painters, while inheriting the essence of tradition, continue to summarize and search for a painting language in line with the atmosphere of the times, gradually shifting their perspective to a separate, tiny “corner” of life, recording the painter's own subtle emotional fluctuations through such “tiny” scenes. The approach models services through a context-aware service model and establishes flexible driving relationships between IoT data and services through context and scenario concepts. Moreover, it reduces the amount of business data to be processed by the system through contextual events. The process of the approach for service development is described, and relevant context-aware service development tools are introduced. Finally, the feasibility of the approach proposed in this article is illustrated by a service development example. An event-triggered sensor node clustering algorithm based on beamforming data transmission technology is proposed, which can improve network energy efficiency and clustering success rate. Meanwhile, a clustering algorithm based on the energy consumption rate of sensor nodes is designed to optimize the task allocation and scheduling of mobile node access, reduce the node charging delay, and enable the sensor nodes in the network to get energy replenishment in a timely and reasonable manner according to their own energy consumption rate demand.

## 1. Introduction

In terms of subject matter expression, the works are mainly based on expressing the most familiar and well-known things and people in daily life; figure painting takes fashionable life as the main material and tries to reveal people's inner world through painting [1]; flower and bird works also gradually expand the scope of subject matter to still life, daily objects, and fictional fantasy content, with “surrealism” in the selection of subject matter. Through this way of expression, the painter constructs a nonlogical interweaving of history and space-time to reflect the painter's personal feelings [2]. The use of color is often mainly in gray tones, and the painter's creative perspective and starting point are gradually changing, either a panoramic view from afar, or a subtle corner near, giving the viewer a space for imagination, and this gray tone is also in line with the current aesthetic trend [3–6]. In terms of technique, the tools of painting have become more and more abundant with the development of society, and the processing of picture effects has broken the traditional “three alum and nine dye” pattern, weakening the lines, and adopting new techniques such as washing and rubbing, spray painting, and paper rubbing to shape richer picture effects; some foreign pigments have gradually appeared in the picture, such as gouache [7]. These new changes have distinctive characteristics of the times and create an emotional atmosphere different from that of the ancients [8].

In today's highly urbanized society, we are in a complex and complicated society where everyone is an independent individual, and the differences in living environment, education level, and ideology have caused the differentiation of personalities [9]. People are busy in the “castle” made of steel and concrete every day. Although the metropolis brings people convenience and a colorful lifestyle, there is also serious mental pressure along with it [10]. Faced with the rapid development of urbanization, people's lifestyle has become more and more programmed and boring, and people increasingly prefer to confine themselves in a hidden space, begging for a trace of inner peace. In different social environments and different cultural backgrounds, what painters want to express naturally differs. Contemporary painters no longer express their respect for nature as traditional paintings do, but, instead, they are able to portray their own ideas and express their own unique experiences and feelings [11]. The painter changes his perspective to start from his daily life and harmoniously integrates the common things in real life into his own picture through subjective processing, and his creation is more and more focused on expressing subjective emotions, with more emphasis on living and anthropomorphism in his works [12]. The IoT architecture usually includes three layers: the perception layer, the network layer, and the application layer [13]. The perception layer is the core of the IoT architecture, which mainly identifies and senses the surrounding environment through sensors and other application technologies and collects the required data information to achieve wide-area perception [14]. The network layer is mainly responsible for data exchange and transmission; i.e., the information collected by the perception layer is transmitted by network technologies, such as virtual private network, wireless communication network, wireless LAN, and telephone exchange network, and secure and reliable communication is realized by key technologies such as near-range wireless communication technology, heterogeneous network convergence access technology, and IP carrier technology [15–18]. The application layer uses the collected data to combine the information needs of individuals, families, and industries and provides rich information services for different users through external interfaces [19].

Implementing caching in IoT scenarios requires addressing multiple challenges, among which the key challenges include the following three aspects. (1) Freshness of IoT data: due to the freshness of IoT data, caching IoT data are more challenging than caching traditional Internet data because the lifecycle of IoT data and its freshness need to be considered when making caching decisions; (2) dynamic nature of IoT data. Since the popularity distribution of IoT data may change with time and location, corresponding popularity prediction schemes need to be designed to more accurately cache the files that users may request; (3) user mobility: in IoT scenarios, since most services will be delivered to mobile users, and when mobile devices move from one base station with caching power to another service interruption may occur, how to guarantee the complete data transmission to the user in mobile is the key to IoT services.

The approach models services through a context-aware service model, establishes flexible driving relationships between IoT data and services through context and scenario concepts, and reduces the amount of business data to be processed by the system through contextual events. The process of the approach for service development is described, and relevant context-aware service development tools are introduced.

## 2. Reconstruction of IoT Sensing Service Nodes in a Brushstroke Style

### 2.1. Scene Construction Based on the Freshness of Brushwork

The traditional brush painting focuses on white space and pays attention to the relationship between main and secondary, sparse and dense, interspersed and other changes, and the composition of the picture is vivid and simple [20]. Contemporary painters absorb the composition characteristics of traditional painting, expand and study the pattern, and make the composition form colorful; they pay attention to the expression of space and structure when composing, and the composition is extremely simple, such as a flower, a lamp, a glass, a meaningful piece of cloth, a pair of high heels and other living objects; or the desktop, windowsill, and other scenes of modern life are all skillfully arranged in the whole picture. These things are arranged in a beautiful context with a fine and clever layout of the picture space, or one of the objects is exaggerated in close-up proportion to emphasize the focus, and the subject is depicted in a very delicate and subtle way. From the perspective of the arrangement of objects in the picture, each individual item is mostly in a static state in the picture or cleverly combined in the picture. Most of them are in the middle position, attracting the viewer's eyes and presenting a symmetrical relationship visually. Such a composition gives a sense of quietness, stability, and balance, and such a set is particularly easy to attract the viewer's attention while having a distinctive period. The network layer is mainly responsible for data exchange and transmission; i.e., the information collected by the perception layer is transmitted by network technologies such as virtual private network, wireless communication network, wireless LAN, and telephone exchange network.

**Figure 1:**
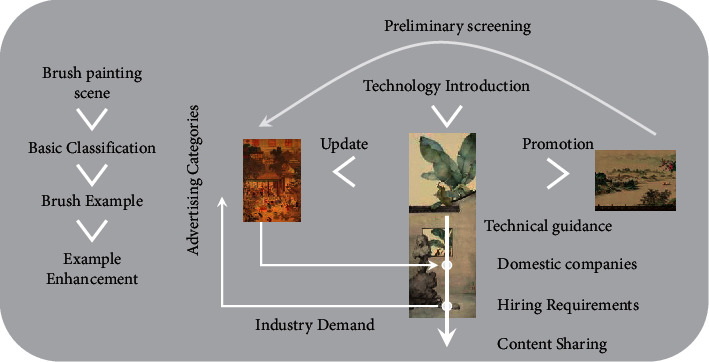
Brush painting scene system model.

The system model in this article is shown in Figure 1, which consists of IoT data source, Internet, *I* small base stations, and *U* users, respectively, where *I* = {1, 2,…, *I*}, *U* = {1, 2,…*, U}*, and *{I* + *1}* denote the small base station set, the user set, and the Internet, respectively. In the model of this chapter, each small base station has a limited cache capacity, and a small base station can serve multiple users, and its corresponding set of cache capacity is as follows:

(1) $$\begin{array}{c} S=\left\{s_{1},s_{2},\ldots ,s_{n}\right\}. \end{array}$$

There are J IoT data files in the IoT data source, and the set of all data files is as follows:

(2) $$\begin{array}{c} Q=\left\{q_{1},q_{2},\ldots ,q_{n}\right\}. \end{array}$$

The corresponding set of file sizes is as follows:

(3) $$\begin{array}{c} U=\left\{u_{1},u_{2},\ldots ,u_{n}\right\}. \end{array}$$

And the Internet obtains real-time files from data sources and provides them to each small base station. In addition, the small base stations communicate with each other through wireless links, so they can establish communication with each other only when the distance between the small base stations is within a certain range, so each small base station has a set of associated connections, denoted as the following set:

(4) $$\begin{array}{c} T=\left\{t_{1},t_{2},\ldots ,t_{n}\right\}. \end{array}$$

Other small base stations can establish connections with each other, i.e., *Si* ⊆ *l*.

In addition, the small base station unit download costs must be lower than the Internet unit download cost, as shown in Table 1:

(5) $$\begin{array}{c} D_{i,j}^{g}<D_{i,j}^{g+1}, \end{array}$$

and for the same file *j, D* and *T* cannot appear at the same time.

### 2.2. Service Scenario Node Collaboration Strategy

Usually, the request service time refers to the time when the small base station transmits the file to the user through the downlink or when the small base station downloads the file from the Internet and then transmits it to the user through the backhaul link, which is negligible compared to the time when the file is cached in the base station. Contemporary brush painting “microscene” works are extremely simple, often appearing as independent objects so that the composition looks as if space and time have stood still, giving people a sense of ethereality.

This article also introduces relevant concepts in the IoT scenario. IoT data are usually valid for a specific period of time after the data are generated, which is defined as the data lifetime and is defined by the service provider. In this chapter, *F*_*j*_ is used to denote the lifetime of file *E*_*j, i, j*_ denotes the age of file j cached in base station *i*, and each file contains a lifetime value. In addition to this, some users have stricter restrictions on the age of some files, such as real-time requirements of temperature APP for temperature information and real-time requirements of traffic recorder for traffic light information. Therefore, part of the user's request contains a freshness parameter related to the age of the file, which is defined in this article as the freshness requirement of user *u* for file *j*, denoted by *F*_*u, j*_. In this article, *F*_*u*_ is a stricter parameter than *F*_*j*_, i.e., for any file *j* satisfying

(6) $$\begin{array}{c} F_{i,j}^{g}<F_{i,j}^{g+1}. \end{array}$$

From the definition, it is clear that not all requests contain *F*_*u,j*_ values, and the *F*_*u,j*_ values contained in the request are different for different users requesting the same file due to different user requirements. When a user receives a file from a base station, the user will get a value based on the age of the file. If file *j* from small base station *i* is passed to user *u*, the value of the file obtained by the user at this time is defined as follows:

(7) $$\begin{array}{c} A_{i,j}=\left\{A^{\max}\left(\frac{E_{i,j}-1}{3}\right),E_{i,j}\right)<F. \end{array}$$

From the definition of file value, it can be seen that the value of the file obtained by the user will decrease with the age of the file. When the age of file *j*, *E*_*i, j*_, does not exceed the survival period *F*_*j*_, the value of the file decreases slowly with the age of the file, while when the age of file *E*_*i, j*_ exceeds the survival period *F*, the value of the file obtained by the user will decrease sharply with the increase of the age of the file. In addition, the user will get the maximum value of the file when the file stored in the core network is passed to the user, i.e., when the file age is 0.

(8) $$\begin{array}{c} g_{i,j}=\left(1-M_{i,j}\right)*f_{i,j}*v. \end{array}$$

Therefore, when the value of these three factors of small base station *k* is larger, the download cost paid by small base station *i* to small base station *k* will be higher. At this point, the three parameters *c(k), d(k)*, and *m(k)* are normalized to obtain *c(k), d(k)*, and *m(k)*, as shown in the Table 2, i.e., *c(k), d(k)*, and *m(k)* ∈ [0, 1]. These three factors are then combined by their respective weighting factors *α, β*, and *γ* and have *α* + *β* + *γ* = 1. The final download cost when small base station *i* requests a file from small base station *k* is as follows:

(9) $$\begin{array}{c} H_{i,j}=D_{i,j}^{k}*v*\frac{1}{ac\left(k\right)+\beta \;d\left(k\right)+\gamma m\left(k\right)}. \end{array}$$

**Figure 2:**
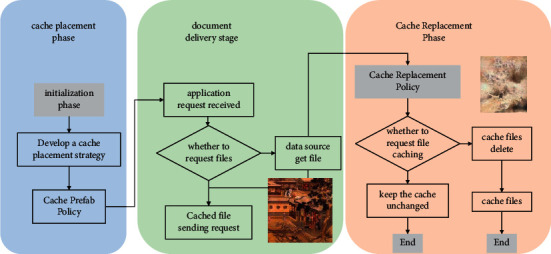
Access to files by small base stations.

It can be seen that the smaller the values of *c(k), d(k)*, and *m(k)*, the higher the collaboration potential of the associated small base station *k*. In other words, when a small base station *i* cannot satisfy the user's demand, the possibility of having a small base station *k* to provide the files required by the user is higher. In this case, the download cost is smaller when small base station *i* requests files from small base station *k*. From the above analysis of the two costs, it can be seen that when the connected small base station *i* of service user *u* does not have the file *j* required by the user cached, small base station *i* can obtain the file through the Internet or any associated small base station *k*. Four different cases arise, as shown in Figure 2. While the term “freshness” indicates how recent the IoT data have been generated, this chapter uses the metric of file age to describe freshness, i.e., the duration of data from its generation to the current moment.

A sensor node that detects an event and needs to transmit information is called a trigger sensor node. If the number of nodes in the communication range has reached the demand for the number of beamforming nodes, then the clustering occurs within the communication range of the trigger node. If the number of nodes within the communication range of the trigger node does not reach the required number of beamforming nodes, the trigger node sends a request message to the outside of the cluster starting from the promoted cluster head to request collaborative beamforming. After the successful clustering, the trigger node transmits the collected information to its parent node (cluster head), which sends it to other cluster heads according to the transmission path, and then other cluster heads send it to their children to achieve the purpose of data sharing and synchronization. The network, after clustering by this algorithm, has the following characteristics:

1. To prevent the number of nodes in a single cluster from falling short of the need for beamforming long-range communication, intercluster heads need to be communicable to obtain more nodes to collaborate on beamforming.
2. Adopting a circular dispersion structure suitable for phase synchronization and controlling the number of layers to three.
3. The number of nodes within the communication range of the trigger node is counted before the algorithm starts, and the number of nodes is predicted in advance to avoid unnecessary loss of energy due to clustering failure caused by an insufficient number.
4. The promoted cluster head nodes give priority to nodes at one-half of the communication distance of the trigger node so that the cluster head and its subnodes contain all nodes that trigger the node statistics before the algorithm is executed, which avoids the failure of clustering to some extent.
5. To save energy, clustering uses only some of the nodes in the network and there exist nodes that are neither cluster heads nor subnodes. Based on the number of nodes in the communication range, the trigger sensor node determines whether it needs other nodes to collaborate and request more nodes to join the cluster.

**Figure 3:**
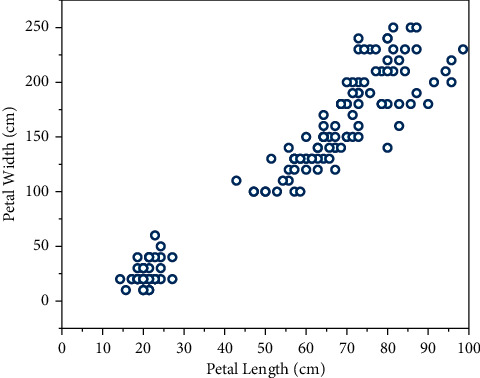
Simulation results for 200 nodes.

Figure 3 shows the clustering results of spreading 200 nodes selecting 6 cluster heads (6 iterations) in a 250 × 100 cm^2^ scene area, respectively. From the figure, it can be seen that using this algorithm in the case of a large number of sensors spreading, only the nodes relatively close to the trigger point are selected to collaborate in beamforming and uploading information, avoiding the waste of resources caused by starting too many nodes and prolonging the network lifetime. The number of nodes within the communication range of the trigger point already reaches the expected number of nodes (100), so only the cluster head is selected within the communication range and no more nodes are requested to be started by the cluster head.

The perception layer is the core of the IoT architecture, which mainly identifies and senses the surrounding environment through sensors and other application technologies and collects the required data information to achieve wide-area perception.

### 2.3. Cluster Success Probability for Scenario Construction

**Figure 4:**
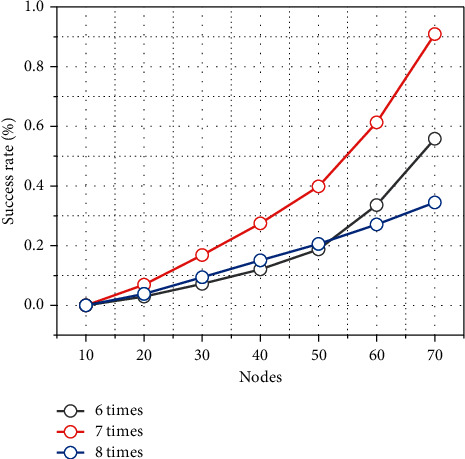
The relationship between algorithm success rate and node distribution density.

Since the sensor nodes in the network are randomly spread, it is inevitable that there will be cases where good communication between the selected cluster heads is not possible due to the failure to gather a sufficient number of nodes. However, the algorithm has taken this aspect into account and tried to reduce the probability of failure. It can be seen that with a sufficient number of nodes, the probability of such failure is still very low.  Figure 4 shows the relationship curves between the success rate of the algorithm and the node distribution density, with 100 trials and the total number of nodes varying from 200 to 500, and the four curves are the results of the algorithm after 6, 7, 8, and 10 iterations, respectively. It can also be seen that the increase in the number of iterations helps to improve the success rate, but the increase in the number of iterations will cause the increase of cluster heads, and too many cluster heads are not conducive to the synchronization and information sharing among cluster heads, so the number of iterations should not be taken too large. At the end of the algorithm, the total number of nodes is counted again, and the decision is made by combining whether the communication between cluster heads is smooth or not.

## 3. Results and Analysis

### 3.1. Simulation and Results

**Figure 5:**
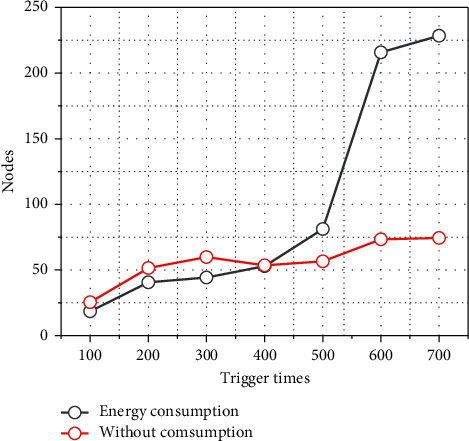
Random energy consumption at trigger points.

In order to extend the lifetime of the whole network, the algorithm is designed with the consideration of node energy. At the same time, in order to improve the success rate of the algorithm and to reduce the influence of the factors that make the clustering fail because the number of nodes cannot reach the expected number, at least 6 iterations are used in the algorithm, and if 6 iterations cannot wake up enough nodes, the 7th, 8th, and 10th iterations are performed. In the simulation, the node energy consumption model is designed based on the process of node communication and data processing in the algorithm to simulate the actual scenario. Next, the relationship between node density and the number of clusters is discussed, as shown in Figure 5. The initial number of nodes is 500, and the two curves are the node density variation curves of the algorithm considering energy factor and the algorithm not considering energy factor, respectively, where the algorithm without energy consideration clusters only based on the distance between nodes. The trigger nodes in the figure are nonfixed and randomly selected in the spreading area. As the number of clusters increases, the node energy is consumed continuously, the nodes start to die gradually, and the node density gradually decreases. The coordinates of the node density of 85 nodes/10000 m^2^ for 95% success power of 6 iterations are marked in the figure as a sign that the whole network stops working. From the simulation results, it can be seen that the algorithm that takes into account the energy balance factor has a much slower decrease in node density and is able to cluster more total times. The uncertainty of node location distribution allows the possibility of cluster failure. If the clustering fails, the cluster head push ratio is adjusted and clustered again.

In addition to beamforming transmission, a mobile node can have one-to-one access to a sensor node if it has access to the sensor network within the sensing area or even within the communication area of each sensor node. This subsection is studied in that case. Sensor nodes in wireless sensor networks are battery-powered and their lifetime is limited by the battery capacity, but with the development of wireless charging technology, a sensor node is no longer available only as a disposable device; it can also be used continuously by recharging energy through this technology. In recent years, the sensor energy supply problem has attracted a lot of attention from researchers. In this case, there are delays in accessing sensor nodes by mobile nodes. Task scheduling of mobile nodes in the network plays a crucial role in achieving high charging and information collection efficiency. During the travel of mobile nodes, some sensor nodes are accessed when they do not need to be recharged, while other sensor nodes that urgently need to be recharged do not receive timely energy replenishment. This not only increases the travel distance of mobile nodes but also prolongs the waiting time of high energy-consuming sensor nodes. It can be seen that the success rate of the algorithm increases gradually with the increase of node density, and the rate of increase decreases until it is close to 1.

**Figure 6:**
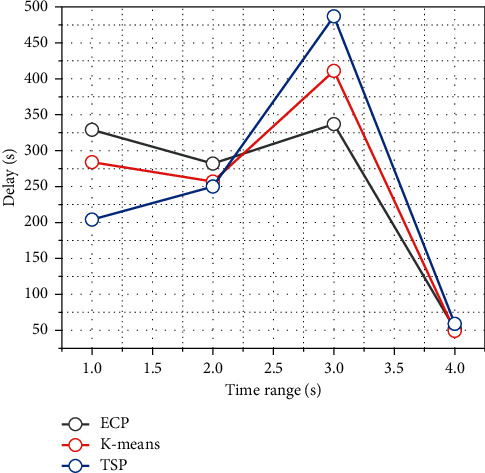
Effect of simulation time on average charging delay.

In this subsection, the average charging delay and the number of visits of ML-ECP, K-means-based *m* TSP, and branch delimitation-based TSP algorithms are given for different sensor node number scenarios. The *K*-means algorithm is a classical clustering algorithm that takes the distance between nodes into consideration, and the K-means-based TSP means that after applying the K-means algorithm to cluster the sensor nodes in the sensing area, the access path of the mobile nodes in each cluster is determined by the branch delimitation algorithm based on the clustering result. The overall mobile node access trajectory for each round of access is obtained by planning the access path of mobile nodes in each cluster based on the branch-and-bound method. In the same access time interval, the three algorithms plan their access trajectories in a scenario with 80 to 160 randomly scattered sensor nodes and a sensing area size of 200 × 200 m^2^, respectively.  Figure 6 shows the average charging delay for the three algorithms' visits. It can be seen from the figure that the average charging delay obtained by each of the three algorithms increases as the number of sensor nodes increases, but the average charging delay obtained by the ML-ECP algorithm planning for mobile nodes is always significantly smaller than the results obtained by the K-means and TSP algorithms planning, and it can be seen from the figure that the ML-ECP algorithm always has the highest number of successful visits accordingly. Thus, it can be shown that the ML-ECP algorithm is more efficient in charging accesses within the same access time interval.

**Figure 7:**
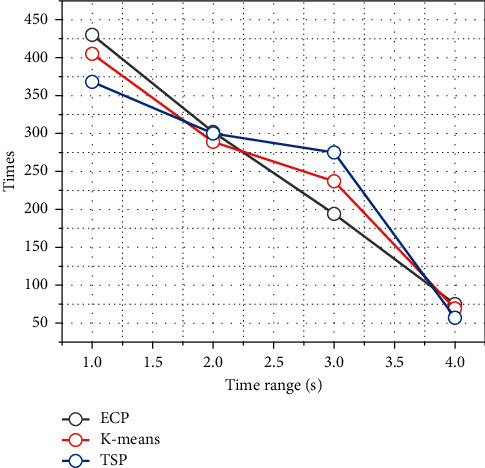
Effect of simulation time on the number of successful visits.

In order to verify the sustainability of the algorithms, this subsection analyzes the effect of given different time intervals on the average charging delay and the number of visits of the mobile nodes, and the simulation results are shown in Figure 7. From the figure, it can be seen that the average charging delay obtained by these three algorithms separately does not change much as the running time increases, which indicates that the mobile nodes can be stable in the network to complete the access tasks by the planning of these three algorithms separately. Among them, the average charging delay obtained by the ML-ECP algorithm proposed in this chapter is always significantly lower than that of the K-means-based mTSP algorithm and the branch-and-bound method-based TSP algorithm. Through the planning of the three algorithms, although the number of sensor nodes successfully accessed by mobile nodes is increasing, the ML-ECP algorithm not only has a smaller average access delay than the other two algorithms but also has a higher number of successful accesses than the other two algorithms. This indicates that the ML-ECP algorithm can enable the mobile nodes to complete the charging access task consistently and efficiently. The planning of travel trajectories of mobile nodes and controlling the occurrence of the network energy hole problem are among the hot topics of research in recent years. Most previous work has focused on the distribution of sensor nodes and information transmission requirements while ignoring the different energy consumption rates and energy requirements of each sensor node.

The beamforming approach can be randomly clustered with high adaptive capability, but the process requires a large number of sensor nodes to collaborate in the process, which is more suitable for application environments with low information transmission frequency. In this chapter, based on the analysis of these characteristics of the beamforming scheme, an event-triggered sensor node clustering algorithm is proposed to manage the sensor nodes in the network and improve the clustering success rate. This chapter focuses on the problem of uneven energy replenishment demand of sensor nodes in the network and proposes a clustering algorithm based on the energy consumption rate of sensor nodes. By improving the fuzzy algorithm in the machine learning algorithm, the sensor nodes in the network are clustered according to the variability of the energy consumption rate of sensor nodes, so that the sensor nodes can get energy replenishment on demand, reasonably and timely, and reduce the node charging delay.

### 3.2. Real-Time Scheduling

The HMDG algorithm designed in this chapter is compared and analyzed with the solutions of the branch-and-bound-based TSP algorithm and the K-means-based mTSP algorithm under different scenarios of parameter values. The TSP algorithm based on the branch-and-bound algorithm is to solve it directly using the branch-and-bound algorithm. The K-means-based mTSP algorithm directly converts the mTSP into a TSP using the K-means algorithm and then solves the TSP in each cluster using the branch-and-bound method. In order to analyze the advantages of the two clustering algorithms in the second step of HMDG and the two path planning algorithms in the third step under different scenarios, they are compared and analyzed in different scenarios. All results are the average of 100 simulation experiments. Since the simulation time of the TSP algorithm in the comparison algorithm is too long for more than 100 sensor nodes, in order to shorten the simulation time, this section conducts simulation verification and comparison analysis in an area of 200 × 200 m^2^ with 100 sensor nodes randomly distributed. Four mobile nodes outside the sensing area are fully charged from the aggregation node and periodically visit the sensor nodes in the sensing area according to their access tasks. The mobile nodes are assumed to move at 1 m/s. The battery capacity of the sensor nodes is *E*0 = 0.5 J and the path loss index is *α* = 2. The scheme of mobile node access allows the mobile nodes to penetrate deep into the sensing area of the sensor nodes and can replenish the energy of the sensor nodes while collecting information with the help of wireless charging technology.

**Figure 8:**
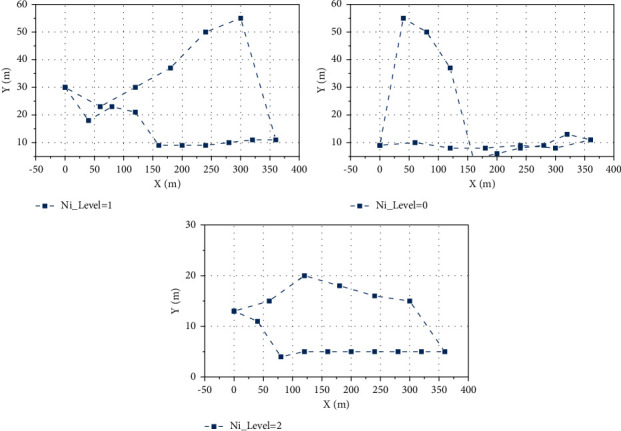
Verification of IoT-based relaxation algorithm.

Among them, Figure 8 shows the results of assigning all sensor nodes in the network to the four levels after the first step of the HMDG algorithm. In Figure 8, red asterisks indicate that some sensor nodes have been assigned to Ni_level = 2; blue hollow circles indicate that some sensor nodes have been assigned to Ni_level = 3; sensor nodes at Ni_level = 1 as child nodes are marked by yellow square triangles. Since there are no sensor nodes assigned to Ni_level = 0 in this example, there is no Ni_level = 0 marker.

**Figure 9:**
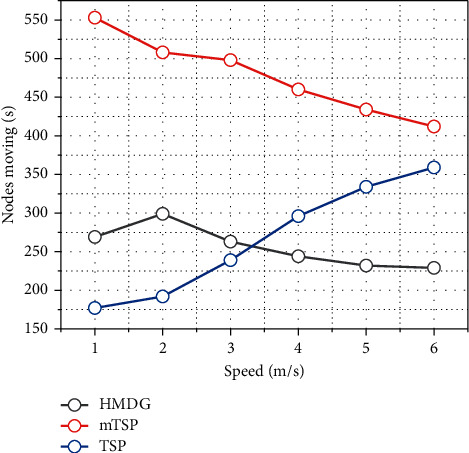
Number of nodes moving at different speeds.

In order to verify the relaxation algorithm in the third step of HMDG, 10, 25, and 50 sensor nodes are deployed in a single cluster in this subsection. Based on the same initial sensor node locations, the trajectories of the mobile nodes are planned using the branch-and-bound method and the relaxation algorithm designed in this article (when *r* = 0), respectively, and the results are shown in Figure 9. The branch-and-bound method is a classical solution to the integer programming problem that is considered to be nearly exact. The top three figures depict the planning results of the branch-and-bound method, and the bottom three figures depict the planning results of the relaxation algorithm proposed in this chapter. Since the branch-and-bound method can only solve the integer planning problem, it cannot plan the trajectory of the mobile node for the mixed-integer problem of finding the convergence point within the threshold of wireless communication in this chapter, so the communication radius is set to *r* = 0. Then, the two algorithms are compared. It can be seen that the results obtained by both algorithms satisfy all the constraints, the mobile node visits each sensor node once and only once, and the paths chosen by both methods are almost identical at each step. Thus, the correctness of the algorithms is verified.

Color plays an important role in brush painting. Whether in ancient or modern times, the colors in works have distinctive characteristics of the times. In terms of color style, traditional brush painting is more concerned with “assigning color to the class,” and the painters use less harmonious colors, requiring bright and simple, harmonious and elegant, and rich and elegant colors while paying more attention to the inherent colors of things themselves. By using the inherent color of things, the painter renders them over and over again, striving for the composure of the picture so that the picture achieves a harmonious and unified effect. Moreover, the picture is not affected by external conditions such as special light and environmental colors, and the work avoids bright and gorgeous appearance but empty content without connotation, so the painter also has a certain subjective consciousness in the picture processing and the decorative effect in the picture is more or less reflected.

A comparative analysis of the mobile node trajectory lengths obtained by three mobile node travel trajectory planning algorithms, HMDG based on the relaxation algorithm, HMDG based on the greedy jump algorithm, and mTSP based on K-means, is performed. The simulation results of the number of sensor nodes from 40 to 200 randomly spread in a square area of 200 × 200 square meters, with 4 mobile nodes for data collection and charging of the target area. The effect of network density on the algorithm is investigated by varying the number of sensor nodes in the network. As shown in the figure, as the number of sensor nodes in the network increases, the mobile trajectory lengths of all three algorithms grow. The mobile trajectory lengths of both HMDG algorithms are consistently shorter than the K-means-based mTSP method, and the difference in trajectory lengths increases gradually with the increase in the number of sensor nodes.

## 4. Conclusion

A context-aware IoT service development method is proposed to address the problem that traditional web service development methods, such as traditional software engineering methods or service-oriented architecture, do not adapt to the flexible and volatile IoT environment with huge data volume. The approach models services through a context-aware service model, establishes flexible driving relationships between IoT data and services through context and scenario concepts, and reduces the amount of business data to be processed by the system through contextual events. The process of the approach for service development is described, and relevant context-aware service development tools are introduced. Finally, the feasibility of the approach proposed in this article is illustrated by a service development example. An event-triggered sensor node clustering algorithm based on beamforming data transmission technology is proposed, which can improve network energy efficiency and clustering success rate. Meanwhile, a clustering algorithm based on the energy consumption rate of sensor nodes is designed to optimize the task allocation and scheduling of mobile node access, reduce the node charging delay, and enable the sensor nodes in the network to get energy replenishment in a timely and reasonable manner according to their own energy consumption rate demand. A heuristic mobile node trajectory optimization algorithm is proposed using an offline scheduling model with multiple mobile nodes as relays. The algorithm can optimize the allocation and scheduling of data collection and charging tasks among multiple mobile nodes, shorten the trajectory length and data collection delay of mobile nodes, and effectively avoid the occurrence of the network hole problem caused by sensor nodes not getting timely energy replenishment.

The two algorithms are compared. It can be seen that the results obtained by both algorithms satisfy all the constraints, the mobile node visits each sensor node once and only once, and the paths chosen by both methods are almost identical at each step.

## Figures and Tables

**Table 1:** Internet download and caching costs.

Parameters	*D*	*T*	*k*
1	8.61	5.13	3.39
2	9.40	5.27	3.68
3	9.96	4.99	3.32
4	7.66	3.74	2.51
5	9.39	4.59	3.22
6	9.21	4.86	3.31
7	8.53	4.74	3.03

**Table 2:** Variation of IoT base station parameters.

Influencing factors	*c(k)*	*d(k)*	*m(k)*
a	5.90	7.87	3.57
b	4.73	6.87	3.27
c	6.35	9.40	4.31
d	7.27	10.11	4.87
e	7.33	10.32	5.07
f	6.87	8.91	5.66
g	7.03	9.60	6.01
h	7.09	9.42	6.08

## Data Availability

The dataset can be accessed upon request.

## References

[1] Ge X., Zhou R., Li Q. (2020). 5G NFV-based tactile Internet for mission-critical IoT services. *IEEE Internet of Things Journal*.

[2] Li F., Lam K.-Y., Li X., Sheng Z., Hua J., Wang L. (2020). Advances and emerging challenges in cognitive internet-of-things. *IEEE Transactions on Industrial Informatics*.

[3] Kearney M., Burden K., Schuck S. Disrupting education using smart mobile pedagogies.

[4] Ronchi A. M. E-Learning: How Teaching and Training Methods Changed in the Last 20 Years.

[5] Norouzi Shad M., Maadani M., Nesari Moghadam M. (2021). GAPSO-SVM: an IDSS-based energy-aware clustering routing algorithm for IoT perception layer. *Wireless Personal Communications*.

[6] Awan K. A., Din I. U., Zareei M., Talha M., Guizani M., Jadoon S. U. (2019). Holitrust-a holistic cross-domain trust management mechanism for service-centric Internet of Things. *IEEE Access*.

[7] Puliafito C., Virdis A., Mingozzi E. The impact of container migration on fog services as perceived by mobile things.

[8] Nashaat H., Ahmed E., Rizk R. (2020). IoT application placement algorithm based on multi-dimensional QoE prioritization model in fog computing environment. *IEEE Access*.

[9] Aazam M., St-Hilaire M., Lung C.-H., Lambadaris I., Huh E.-N. IoT resource estimation challenges and modeling in fog.

[10] Gu X., Zhang Z. (2021). IoT security and new trends of solutions. *International Series in Operations Research & Management Science*.

[11] Lv Z., Song H. (2020). Mobile internet of things under data physical fusion technology. *IEEE Internet of Things Journal*.

[12] Yang L., Yao H., Wang J., Jiang C., Benslimane A., Liu Y. (2020). Multi-UAV-enabled load-balance mobile-edge computing for IoT networks. *IEEE Internet of Things Journal*.

[13] Fan Y., Zhao G., Lin X., Sun X., Zhu D., Lei J. One secure IoT scheme for protection of true nodes.

[14] Deng L., Li D., Yao X., Wang H. (2019). Retracted article: mobile network intrusion detection for IoT system based on transfer learning algorithm. *Cluster Computing*.

[15] Rao S., Verma A. K., Bhatia T. (2021). A review on social spam detection: challenges, open issues, and future directions. *Expert Systems with Applications*.

[16] Chen Z., Cui H., Wu E., Li Y., Xi Y. Secure distributed data management for fog computing in large-scale IoT application: a blockchain-based solution.

[17] He D., Chan S., Guizani M. (2018). Security in the internet of things supported by mobile edge computing. *IEEE Communications Magazine*.

[18] Fortino G., Fotia L., Messina F., Rosaci D., Sarne G. M. L. (2020). Trust and reputation in the internet of things: state-of-the-art and research challenges. *IEEE Access*.

[19] Davoli L., Paraskevopoulos I., Campanella C. (2021). Ultrasonic-based environmental perception for mobile 5g-oriented xr applications. *Sensors*.

[20] Sharma T., Satija S., Bhushan B. Unifying blockchian and IoT: security requirements, challenges, applications and future trends.

